# Investigation of Roll-Over Characteristics in Healthy Old Individuals and Patients With Peripheral Artery Disease

**DOI:** 10.1093/geroni/igaa057.684

**Published:** 2020-12-16

**Authors:** Sara Myers, Ganesh Bapat, Jason Johanning, Iraklis Pipinos

**Affiliations:** 1 University of Nebraska at Omaha, Omaha, Nebraska, United States; 2 University of Nebraska Medical Center, Omaha, Nebraska, United States

## Abstract

Atherosclerotic blockages in the leg arteries, cause leg pain with ambulation (called claudication), impair gait and substantially reduce the walking ability of patients with peripheral artery disease (PAD). Ankle-foot orthoses are being developed and applied so patients can walk more and with less pain. Roll-over shape (ROS) is a potential design objective for such assistive devices. In the proposed work, we study roll-over characteristics in patients with PAD and healthy older subjects. Gait data of ten healthy older individuals (Age: 72.8 ± 5.5 years) and twenty patients with PAD (Age: 64.1 ± 6.6 years) were collected at self-selected walking speed. In patients with PAD, gait data were collected before the onset of pain and after claudication pain was induced. To generate ROS, the center of pressure data was transformed to the shank-based coordinate system and circular arcs were fit using an optimization program in MATLAB. Independent t-tests with Bonferroni corrections were used to separately compare roll-over radius differences (p<0.05) between healthy older to both walking conditions in patients with PAD. The mean roll-over radius was not significantly different between healthy older vs PAD pain-free (p=0.468) or PAD pain-induced (p=0.289) walking conditions. Our results indicate invariance of ROS radius in patients with PAD, which is consistent with previous literature showing general invariance of ROS in healthy young individuals. Previous biomechanical studies show gait kinematics and kinetics are more affected by PAD than by age. Future studies should focus on the potential adaptive mechanisms in patients with PAD achieving invariant ROS.

